# Low-Cost 3D-Printed Mazes with Open-Source ML Tracking for Mouse Behavior

**DOI:** 10.1523/ENEURO.0141-25.2025

**Published:** 2025-09-24

**Authors:** James D. O’Leary, Dhwani C. Gondalia, Molly O’Brien, Miles Morlock, Gemma Haney, Bevan S. Main, Mark P. Burns

**Affiliations:** Laboratory for Brain Injury and Dementia, Department of Neuroscience, Georgetown University Medical Centre, Washington, DC 20057

**Keywords:** 3D printing, behavior, learning and memory, machine learning, open source

## Abstract

Behavioral neuroscience research often requires substantial financial investment in specialized equipment and software, creating barriers for new investigators and limiting the flexibility of established laboratories. This study explores how 3D printing and machine learning can be combined to reduce startup and operational costs while maintaining research quality. Using 3D printing, we designed and manufactured a mouse T-maze and elevated plus maze to assess cognition and anxiety-like behaviors in male mice. These custom-built mazes demonstrated comparable efficacy with commercial alternatives while offering greater affordability, scalability, and customization. To complement the hardware, we integrated machine learning for automated tracking and analysis of mouse behavior, achieving accuracy equivalent to commercial solutions or experienced human scoring at significantly reduced cost. By combining 3D printing with machine learning, our approach significantly lowers financial barriers for new investigators and enables established research groups to allocate resources more effectively. This approach not only expands research possibilities for established labs but also lowers the barrier to entry for early-career scientists and institutions with limited funding.

## Significance Statement

Behavioral neuroscience often requires costly specialized equipment, limiting accessibility for new researchers and constraining established laboratories. This study presents an innovative low-cost, scalable solution by integrating 3D printing and machine learning for behavioral testing. We developed and validated 3D-printed mazes for cognitive and anxiety-like behavioral assessments, demonstrating performance equivalent to commercial alternatives at a fraction of the cost. Additionally, we employed open-source machine learning algorithms for automated behavior tracking, reducing the need for expensive proprietary software. This approach reduces financial barriers and enhances experimental customization and reproducibility.

## Introduction

Neuroscience relies on specialized equipment and software to study cognition, emotion, and behavior. However, the high cost of acquiring and maintaining such cutting-edge tools poses significant challenges, especially for early-career investigators and departments with constrained budgets (Woolston, 2023). Innovative solutions such as 3D printing and machine learning are transforming research and expanding experimental capabilities (Vora and Abramson, 2020). 3D printing is an additive manufacturing process where objects are constructed layer by layer (Zastrow, 2020). The adoption of 3D printing has significantly enhanced manufacturing for many industries, including research (Berman, 2012; Attaran, 2017). The ability to rapidly produce custom components at a fraction of the cost offers many advantages. As a result, 3D printing has become an invaluable tool for many research institutes (Thiong’o et al., 2021; Withers et al., 2024). Previous studies have demonstrated the utility of 3D printing across a range of behavioral paradigms, including both standard and novel maze designs (Porter et al., 2021). It has also been applied to novel object recognition, allowing researchers to standardize objects and object features across experiments. 3D printing has also been used to construct components of operant chambers, including food reward delivery systems and nose-poke head entry detectors (Mazziotti et al., 2020; Escobar et al., 2022). Open-source designs for an elevated plus maze (EPM) have also been made publicly available (OnTheBrink, 2023). By publishing 3D design files alongside their experimental findings, researchers enable others to replicate experimental apparatuses which enhances rigor and reproducibility (Inayat et al., 2021) helping to overcome persistent challenges in reproducibility within behavioral research (Open Science Collaboration, 2015; Sochat et al., 2016). Overall, open-source 3D printing provides a low-cost, accessible method for students, researchers, and institutions to build high-quality behavioral apparatus. While prior work has demonstrated the potential of 3D printing, few studies have integrated low-cost maze construction with open-source machine learning tools for behavioral tracking. A natural extension of 3D printing behavioral apparatus is the integration with machine learning, which transforms how behavioral data is collected and analyzed. When paired with advanced computational tools, 3D-printed mazes not only reduce overhead but also enable high-precision tracking with automated analysis. Machine learning is enhancing behavior analysis, particularly through the application of deep neural networks that quantify animal behavior with human-level accuracy (Mathis et al., 2018; Valliani et al., 2019). By combing 3D printing for low-cost construction and experimental standardization with machine learning for automated analysis, researchers can significantly enhance both the reliability and depth of behavioral studies while making these methodologies more accessible and resource efficient. However, this powerful synergy remains largely unexplored. Our study aligns with a broader movement toward cost-effective, standardized, and scalable behavioral research by integrating 3D printing and machine learning for behavioral testing. This approach not only expands research possibilities for established labs but also lowers the barrier to entry for early-career scientists and institutions with limited funding.

## Materials and Methods

### T-maze design and fabrication

**Figure 1.**
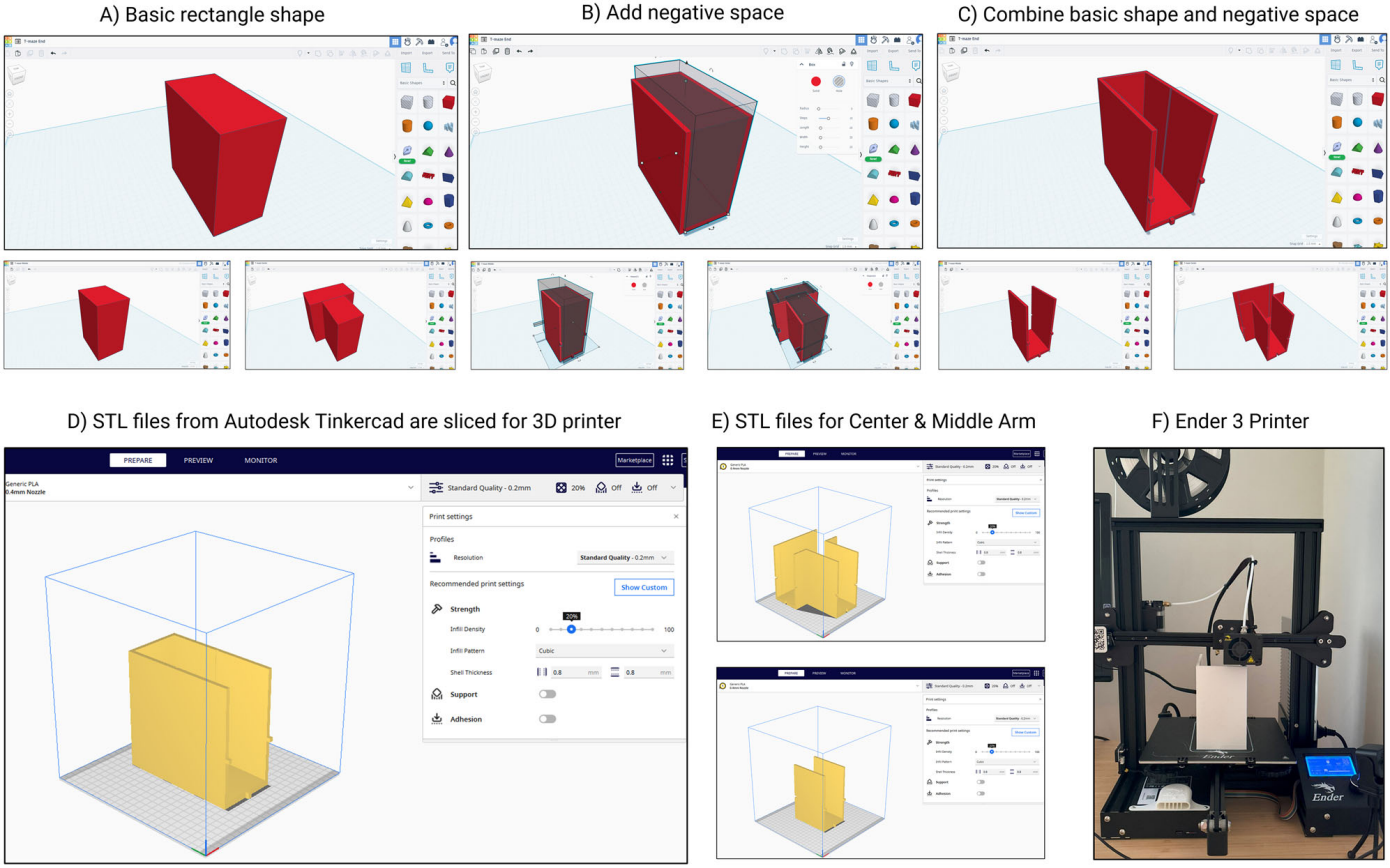
Step-by-step workflow for 3D printing the mouse T-maze. ***A***, A basic rectangular shape is created in Autodesk Tinkercad. ***B***, Negative space is added to define the corridor layout. ***C***, The combination of the rectangle and negative space forms the final T-maze structure. ***D***, The resulting STL file is exported from Tinkercad and imported into slicer software. ***E***, Additional STL files for other T-maze components are prepared for slicing and printing. ***F***, Final printing is performed using an Ender 3 printer.

The T-maze was designed using Autodesk Tinkercad, a free, browser-based platform for 3D modeling (Fig. 1). Tinkercad's intuitive interface and extensive online tutorials make it accessible for users with varying levels of experience (Bryant, 2018). To accommodate the limited print volume of the Ender 3 3D printer, the maze was constructed as modular components (Fig. 1*A–C*). Printers with larger build volumes may allow for fewer sections or even a single-piece print. The final designs were exported from Tinkercad as STL files and imported into Ultimaker Cura (version 5.7.1), a free slicing software used to prepare files for 3D printing (Fig. 1*D*–*F*). Cura's user-friendly interface and robust support resources make it a widely adopted choice for preparing STL files. The maze design files are available on GitHub (https://github.com/BurnsLabGeorgetown/3D_Printing-ML_Neuroscience). All maze components were printed using the Ender 3 with a 0.4 mm nozzle. Polylactic acid (PLA) filament from Creality was selected for its affordability and nontoxicity. Prints were completed with a layer height of 0.2 mm and an infill density of 20%. Each component was visually inspected for defects following printing. The T-maze dimensions: start arm was 34 cm in length and each goal arm 24 cm in length. All T-maze arms were 7 cm width and 15 cm high.

### T-maze assembly

**Figure 2.**
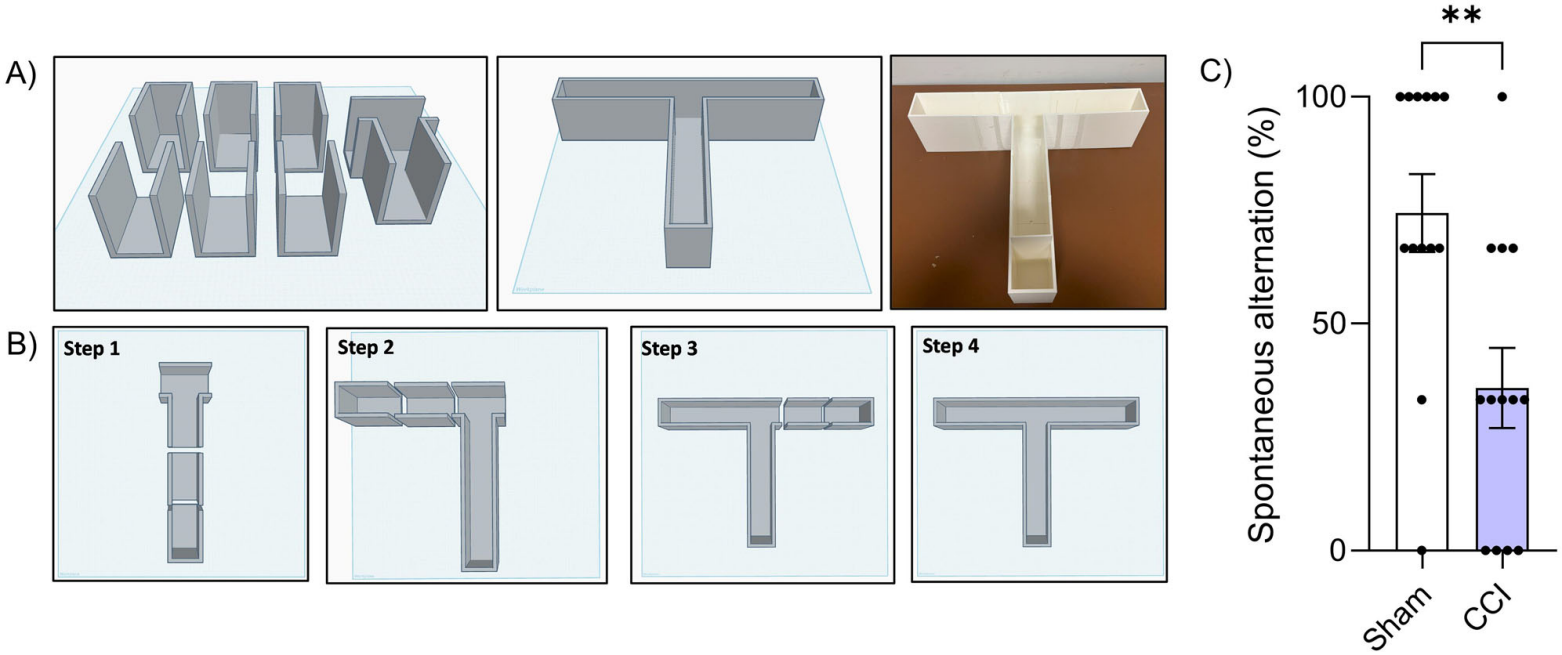
Schematic, assembly, and behavioral data for the 3D-printed T-maze: Schematic diagram of the 3D-printed T-maze design and assembly. Behavioral data from spontaneous alternation. Data are presented as mean ± SEM. Statistical significance was determined using *t* test, with significant differences indicated (**p < 0.01).

Due to the simple rectangular design, all parts printed consistently. Before assembly, each printed section was sealed with a thin layer of epoxy in a two-stage process. First, a foam brush was used to apply epoxy to one side of the maze section. The section was placed on a smaller square mount with a disposable mat underneath to prevent epoxy from running onto the lab bench. After drying for 24 h, the piece was flipped, and the process was repeated to seal the underside. Once both sides were fully cured, the sections were aligned and glued along their edges. They were pressed together until glue was visibly expressed from the seams, indicating a strong bond. Excess epoxy was wiped away immediately with a damp cloth to produce a smooth join. Once fully assembled, a second layer of epoxy was applied to the internal floor of the maze to ensure a robust and uniform seal and a self-leveled surface. The assembled T-maze was then left to dry for another 48 h to allow for the epoxy to fully cure. The T-maze was then cleaned with soap and warm water prior to behavior testing (Fig. 2*A,B*).

### EPM design and fabrication

**Figure 3.**
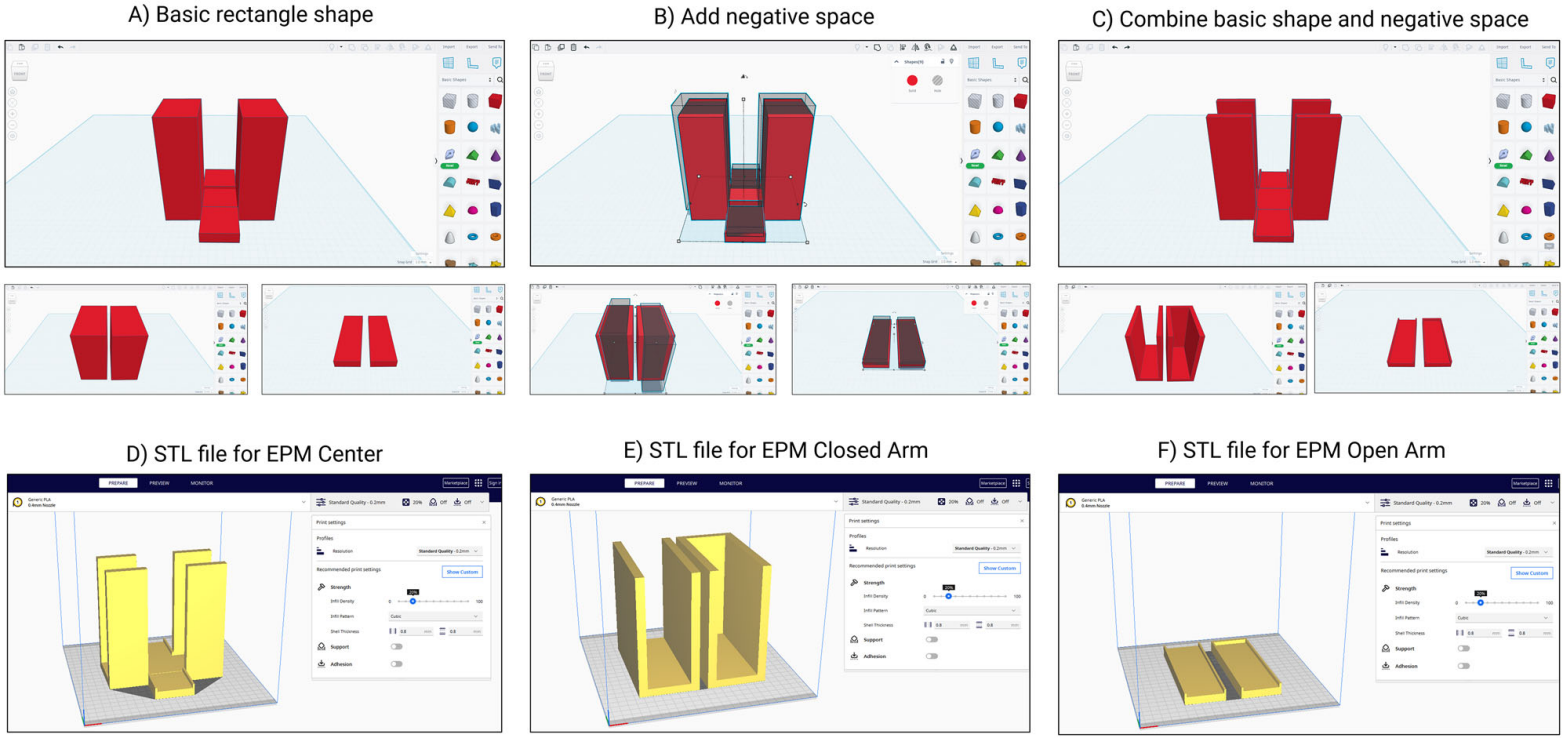
Step-by-step workflow for 3D printing the mouse EPM. ***A***, A basic rectangular and square shape is created in Autodesk Tinkercad. ***B***, Negative space is added to define the open arm and closed arm layout. ***C***, The combination of the basic shapes and negative space forms the final EPM center section. ***D***, The resulting STL file is exported from Tinkercad and imported into slicer software for the EPM center. ***E***, Additional STL files for the closed arm. ***F***, And open arm is prepared for slicing and printing.

The EPM was also designed using Autodesk Tinkercad and similarly constructed as modular components (Fig. 3*A–C*). Again, the final designs were exported from Tinkercad as STL files and imported into UltiMaker Cura (version 5.7.1; Fig. 3*D–F*). The maze design files are available on GitHub (https://github.com/BurnsLabGeorgetown/3D_Printing-ML_Neuroscience). All maze components were printed using the Ender 3 with a 0.4 mm nozzle and PLA. Prints were completed with a layer height of 0.2 mm and an infill density of 20%. Each component was visually inspected for defects following printing. The EPM dimensions: closed arms were 32 × 7 × 15 cm and the open arms were 34 × 5 cm.

### EPM assembly

**Figure 4.**
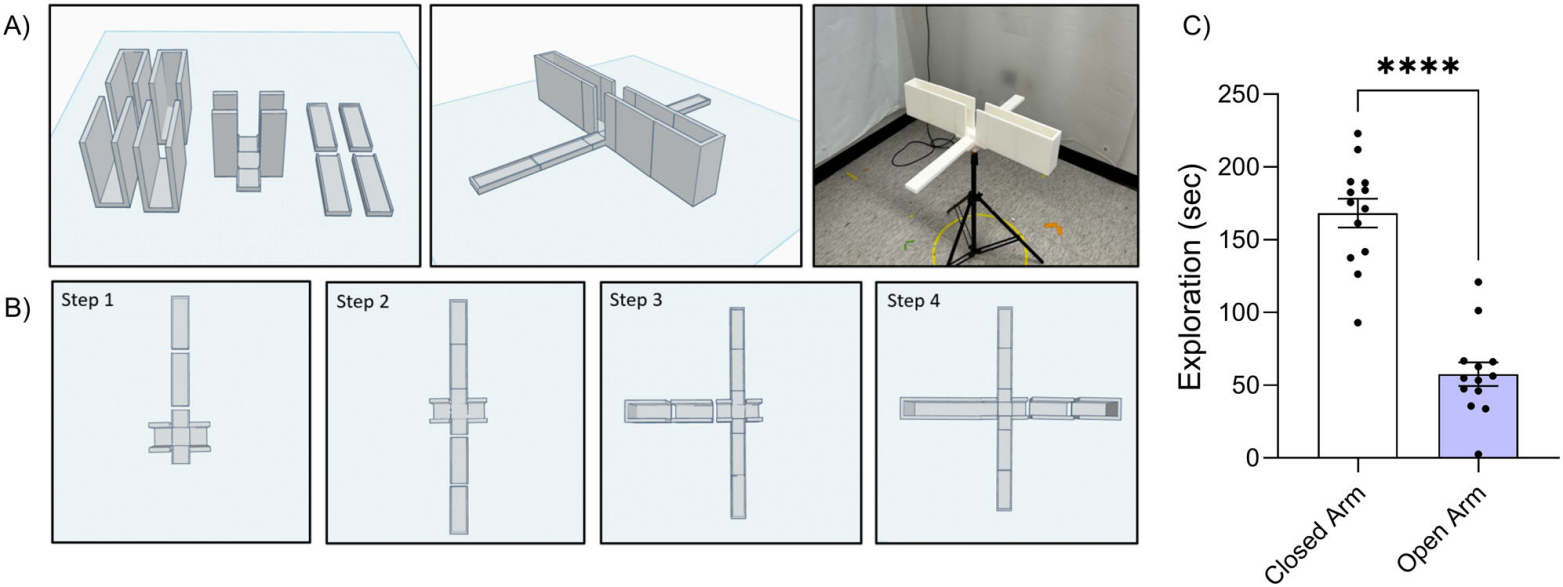
Schematic and assembly for the 3D-printed EPM. ***A***, Schematic diagram of the 3D-printed EPM design and completed apparatus. ***B***, Step-by-step assembly instructions. ***C***, ANY-maze tracking of exploration in EPM.

Following the same procedure as the T-maze, a thin layer of epoxy resin was applied to the surface of each section using a foam brush to facilitate cleaning and create a smooth, sealed surface. The section was placed on a smaller square mount with a disposable mat underneath to prevent epoxy from running onto the lab bench. After drying for 24 h, the piece was flipped, and the process was repeated to seal the underside. Once both sides were fully cured, the sections were aligned and glued along their edges. They were pressed together until glue was visibly expressed from the seams, indicating a strong bond. Excess epoxy was wiped away immediately with a damp cloth to produce a smooth join. Once fully assembled, a second layer of epoxy was applied to the internal floor of the maze to ensure a robust and uniform seal and a self-leveled surface. The assembled EPM was then left to dry for another 48 h to allow for the epoxy to fully cure. Once cured, the EPM was mounted onto a camera stand (Amazon Basics 64 inch Extendable Tripod) to provide the elevated platform (Fig. 4*A*,*B*). One limitation of 3D-printed PLA is its moisture sensitivity, which can degrade strength and surface finish over time. We recommend sealing printed components with a layer of epoxy resin, storing them in dry containers with desiccant, and avoiding prolonged humidity exposure. Gentle cleaning with mild soap and water will also help extend maze lifespan.

### T-maze behavior paradigm

To identify hippocampal-dependent learning and memory deficits, we assessed spontaneous alternation using the T-maze, as previously described (Sloley et al., 2021). Mice were placed in the start area and allowed to acclimatize for 30 s. Once the start door was opened, mice were allowed to enter the start arm and make a choice to enter either the left or right arm. Once inside, the door to the arm was closed and the mouse was confined in the chosen arm for 30 s. Mice were then removed from the maze and returned to their cage while all doors were reset. The test was then repeated. This process was repeated three times on each mouse, with at least 1 h between each session. The number of times that the mice chose the novel arm during the second phase of the trial (spontaneous alternation) was recorded. All testing was performed and analyzed blind to condition.

### Elevated plus maze paradigm

The EPM was used to assay anxiety-like behavior after injury, as previously described (Washington et al., 2016). The 3D-printed apparatus consists of a 26-inch-long cross-shaped platform made of white plastic and sealed with epoxy resin. The EPM was attached to a tripod camera stand with a 3D-printed joint and elevated three feet above the ground. Mice were placed at the center junction of the maze facing an open arm and given 5 min to explore. The time spent in each arm was tracked using the video camera positioned over the maze and analyzed by a trained human observer, machine learning, and ANY-maze tracking software. The camera used was an SVPRO Manual Focus USB Camera, mounted on the ceiling to provide a top-down view of the maze. The camera has a resolution of 1,080 pixels, captures at 30 frames per second, and uses a Sony IMX323 1/2.9 inch image sensor (visible light). The camera angle and focus were manually adjusted to ensure full visibility of the maze and consistent image quality across sessions.

### Control cortical impact injury

The control cortical impact (CCI) surgery was performed as previously described (Loane et al., 2009; Buenaventura et al., 2023). Mice were anesthetized with isoflurane (induction at 3–4% and maintenance at 1–2%) evaporated in oxygen and administered through a nose cone. The head was mounted in the stereotaxic frame and the surgical site clipped and cleaned with alternate iodine and ethanol scrubs. A 10 mm midline incision was made over the skull, and the skin and fascia were reflected to allow a 4 mm craniotomy to be bored on the central aspect of the left parietal bone. A Leica Impact One Stereotaxic Impactor device was used to deliver the cortical impact, with an impact velocity of 5.25 m/s, an impact depth of 1.5 mm, and a dwell time of 0.1 s. After injury, the incision was closed with staples, anesthesia was terminated, and the animal was placed in a heated cage to maintain normal core temperature for 45 min post-injury. Sham-control surgery consisted of exposure to anesthesia, stereotaxic mounting, skin and fascia reflection, and incision closing with staples.

### Animals

Mice were housed in a temperature-controlled room with a 12 h light/dark cycle and food available *ad libitum*. All behavioral testing was conducted during the light cycle. TRAP2xAi32 mice were bred in house. All mice used for the experiments were male between 3 and 4 months of age. Following CCI injury mice were allowed to recover for 1 month before behavioral testing. All mice were grouped housed in standard housing conditions, with temperature 22 ± 1°C, relative humidity 50%, and a 12 h light/dark cycle (lights on 0730 h) and had *ad libitum* access to food and water. All procedures were performed in accordance with protocols approved by the Georgetown University Animal Care and Use Committee.

### Code accessibility

We employed a deep learning-based approach to automate the analysis of EPM behavior using the convolutional neural network package EXPLORE (Ibañez et al., 2023). Originally developed for object recognition tests, EXPLORE is a novel deep learning tool designed to classify exploration behaviors (Ibañez et al., 2023). The Python code and a step-by-step guide to training the model and tracking mouse behavior is available on GitHub (https://github.com/Wahl-lab/EXPLORE). The EXPLORE package includes an intuitive graphical user interface (GUI) for labeling, training, and prediction, allowing researchers to use the software without requiring prior coding expertise (Ibañez et al., 2023). Here we trained the model using a Lenovo ThinkPad with an AMD Ryzen 5 PRO CPU and 16 GB of RAM. In this study, we trained the neural network to track the time spent in each of the four arms of the EPM. The CNN extracts features from video frames and classifies behavior through supervised learning, enabling precise and automated detection of the mouse's location within the maze (Ibañez et al., 2023). A key advantage of EXPLORE is its adaptability by modifying the training dataset, the model can be retrained to recognize behaviors beyond object exploration. Here, we trained the network to differentiate between open and closed arm entries, extending the system's applicability to EPM studies. This flexibility makes EXPLORE a scalable tool for broader behavioral neuroscience applications. Additionally, the model operates efficiently on standard computing hardware, ensuring accessibility for laboratories without high-end GPUs (Ibañez et al., 2023).

### Statistical analysis

All data were analyzed using *t* test or analysis of variance where appropriate, followed by post hoc analysis with the Bonferroni’s multiple-comparison test and presented as the mean ± standard error of the mean. All statistical tests were performed using GraphPad Prism software, version 10 (GraphPad Software).

## Results

To validate the 3D-printed T-maze, we assessed deficits in executive function in a controlled cortical impact model of TBI. Injured mice exhibited significantly reduced spontaneous alternation (35.89%) compared with sham controls (74.36%; *t*_(24)_ = 3.13, *p* < 0.001; 95% −63.84 to −13.08; Fig. 2*C*), indicating impairments in spatial working memory and executive function. This large effect (partial *η*^2^ = 0.29) reflects a robust difference between groups and is consistent with previously published findings (Washington et al., 2012). Together, these results support utility of the 3D-printed T-maze as a reliable platform for detecting executive dysfunction in preclinical models of TBI.

The EPM is a well-established assay for assessing anxiety-like behavior in rodents. In our 3D-printed version of the EPM, mice exhibited a strong preference for the closed arms, spending significantly more time in the closed arms (mean = 168.3 s) than in the open arms (mean = 57.5 s), with an average difference of 110.7 s (*t*_(12)_ = 6.41, *p* < 0.0001; 95% CI: 73.1–148.3 s). This large effect (partial *η*^2^ = 0.77) is consistent with anxiety-like avoidance of open spaces and supports the ecological validity of the 3D-printed EPM (Fig. 4*C*).

**Figure 5.**
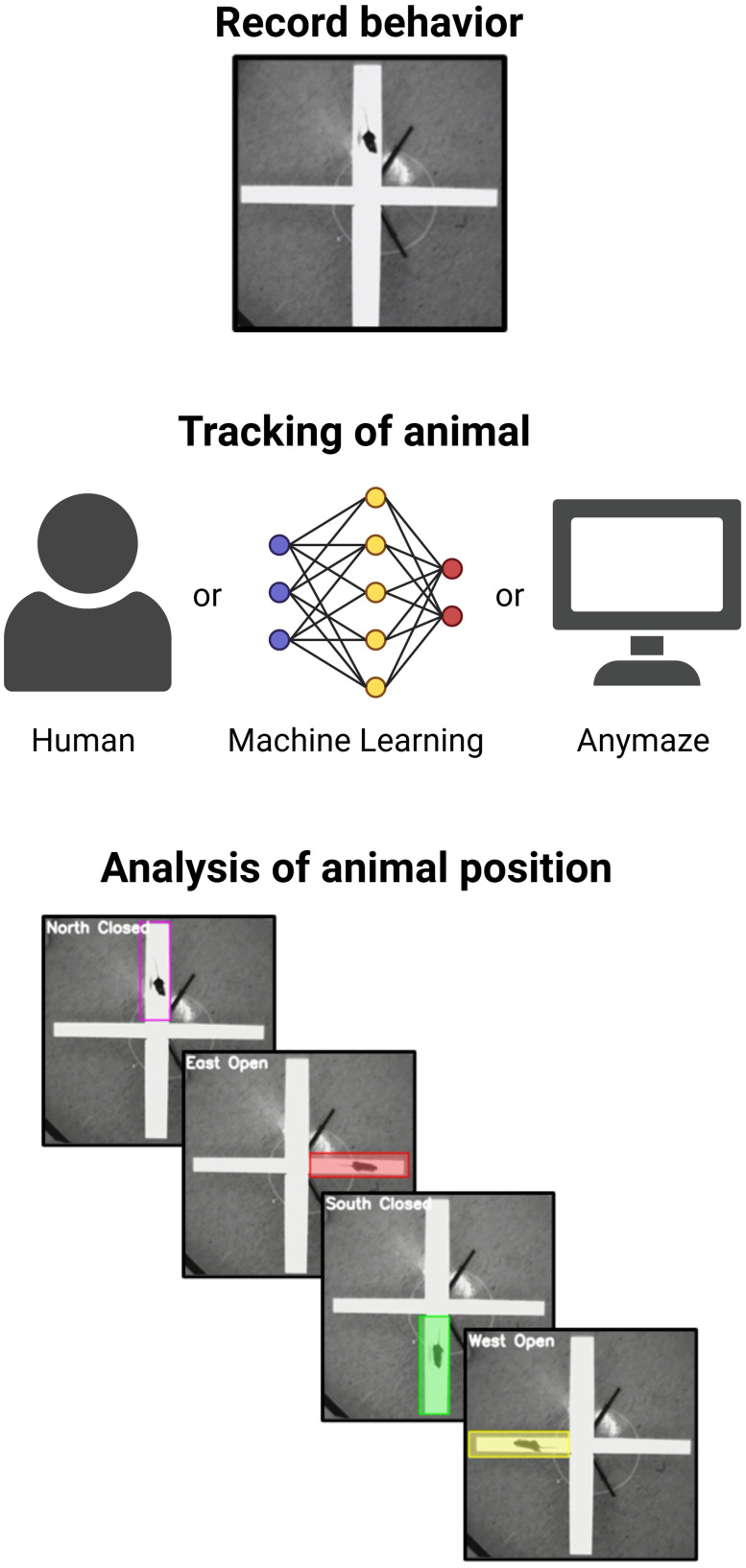
Overview of behavioral tracking and analysis workflow. Illustration of recording, tracking, and analyzing rodent behavior in the EPM. The recorded videos were analyzed by an expert human, automated tracking via deep learning, as well as commercial software ANY-maze. The animal's position within the maze is classified into specific zones: North Closed, East Open, South Closed, and West Open arms.

The analysis of behavior has typically involved labor-intensive manual scoring by a trained expert. Over the years, video tracking software has revolutionized behavioral analysis by enabling precise, automated tracking of animal activity (Vogt, 2021). These tools have provided a user-friendly platform for researchers to analyze parameters such as locomotion, exploration patterns, and time spent in predefined zones (Lim et al., 2023). However, these platforms are often expensive and can be limited in tracking complex behaviors involving subtle or multi-dimensional movements. Addressing these limitations, machine learning-based tools have emerged as transformative alternatives (Vogt, 2021). Using deep learning techniques, trained neural networks can perform highly accurate tracking of an animal's postures and movements, including nuanced and high-dimensional behavior (Mathis et al., 2018; Mathis and Mathis, 2020). This capability significantly enhances the granularity of data collected, broadens the scope of experimental designs, and reduces the time required for manual intervention, offering a clear advantage over traditional software tracking packages. Here we trained a deep neural network for analysis of the EPM and compared the results to human scored data or a commercial software ANY-maze (Ibañez et al., 2023; Fig. 5).

**Figure 6.**
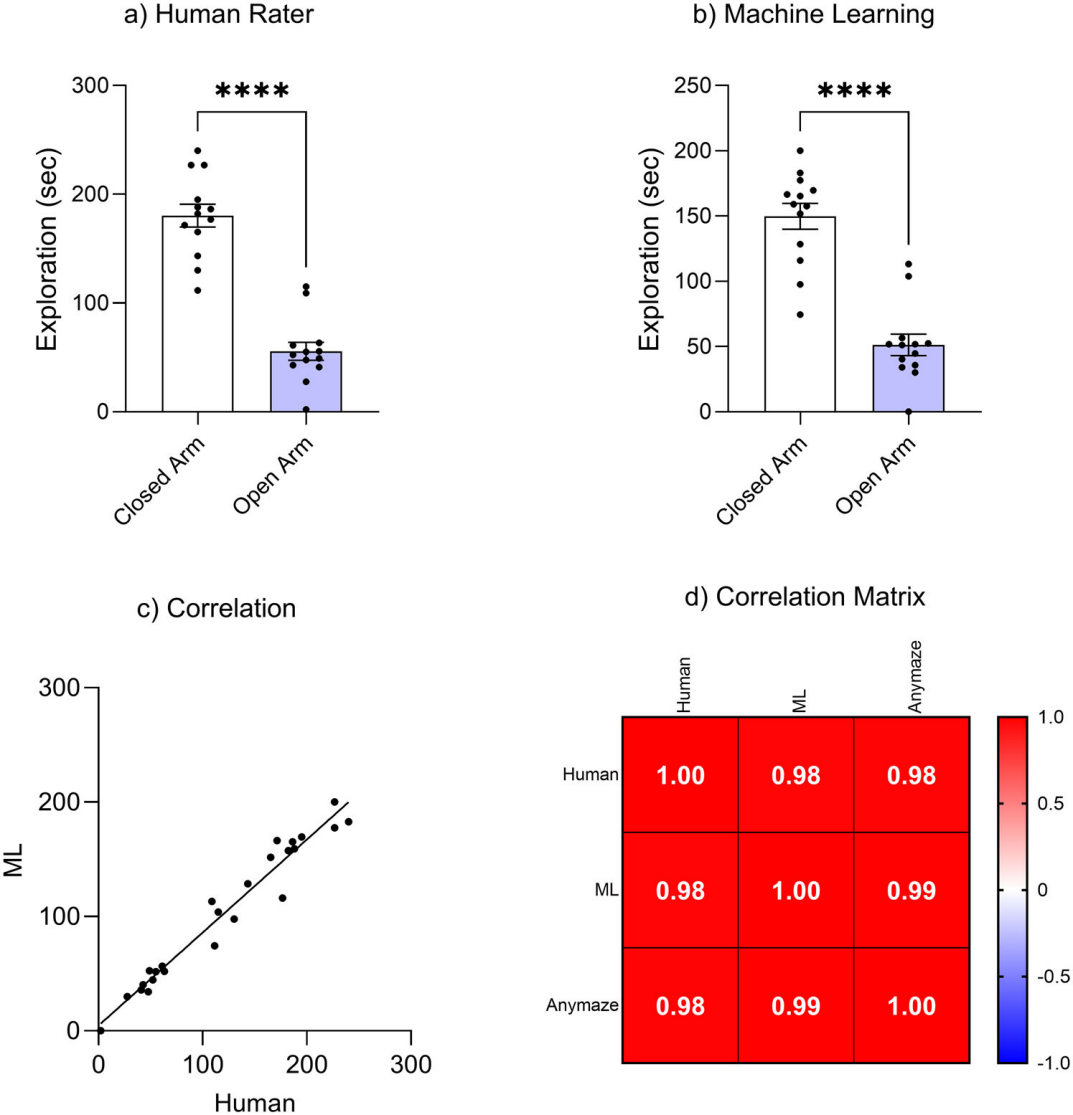
Comparison of behavioral tracking methods. ***a***, Human scored exploration of EPM. ***b***, Machine learning scored exploration. ***c***, Correlation between exploration times scored by the human rater or machine learning. ***d***, Correlation matrix comparing exploration time data across three tracking methods: human rater, machine learning, and ANY-maze.

We compared human trained scoring with machine learning for the EPM. Both human rater manual scoring, (*t*_(12)_ = 7.03, *p* < 0.001) and machine learning-based scoring (*t*_(12)_ = 5.73, *p* < 0.001) revealed a significant preference for the closed arm relative to the open arm (Fig. 6*a*,*b*). This finding is in agreement with the ANY-maze tracking (Fig. 4*C*). Moreover, there was a significant positive correlation between human-based tracking and ML-based tracking, *r* = 0.97, *p* < 0.001, 95% CI [0.95, 0.99] (Fig. 6*c*). Moreover, a strong linear relationship was observed (*R*^2^ = 0.95), demonstrating the reliability of the machine learning method in replicating manual scoring. All methods exhibited high correlation (correlation coefficients ≥ 0.95), indicating robust consistency in behavioral analysis across the different tracking approaches (Fig. 6*d*). This finding highlights the reliability of automated machine learning tools for analyzing rodent behavior, demonstrating strong agreement with manual scoring and commercial software. The results validate machine learning as an accessible and accurate method for behavioral neuroscience research.

## Discussion

This study demonstrates the transformative potential of integrating 3D printing and machine learning as a scalable, cost-effective pipeline for neuroscience research. By designing and fabricating rodent behavioral mazes using 3D printing, and combining this with machine learning tools for animal tracking, we have shown that traditionally expensive commercial systems can be replaced with affordable, customizable alternatives. Using a deep neural network, we achieved accurate and reproducible behavioral quantification comparable with commercial tracking systems or experienced human observers. This automation supports high-throughput data collection and analysis, enabling researchers to expand their research with reduced need for manual quantification. This approach not only reduces startup costs for new principal investigators but also decreases operational expenses for established laboratories. Together, this work demonstrates the utility of the 3D-printed behavioral mazes as a reliable and effective tool for assessing cognitive function in preclinical rodent models.

For early-career researchers, the high cost of behavioral equipment, worsened by inflation, often limits experimental scope (Woolston, 2023). A combined 3D printing and machine learning pipeline lowers these barriers, offering an affordable alternative to traditional tools without compromising research quality. In this project, we printed a T-maze and EPM for just $76.20 in filament and glue, far less than commercial systems, which typically cost $1,000–$2,000 (Table 1). Even when factoring in the purchase of the 3D printer, the total expense remains much lower. Printers can also be shared across labs or housed in core facilities, reducing per-user cost and increasing accessibility. A complete behavioral maze setup using this approach can be built for ∼$700, with the largest cost typically being the computer used for recording behavior. However, the PC cost can be reduced further. Many universities have e-waste programs with unused or outdated computers that are discarded due to expired security support. These machines can be revived with open-source operating systems like Linux and repurposed for task-specific roles such as behavior recording. For researchers starting from scratch, this demonstrates that building a reliable setup does not require buying everything new, cost-effective, and sustainable alternatives are readily available. Emerging recycling strategies further support this approach, enabling failed prints and post-consumer waste to be converted into new print filament (Anderson, 2017; Lanzotti et al., 2019). The broader adoption of 3D printing aligns with growing interest in sustainable research practices (Jain, 2022). As funding tightens, labs must prioritize resource-efficient solutions that reduce waste and support high-quality science.

**Table 1.** Cost breakdown of components used in T-maze and EPM fabrication

Item	Description/application	Cost (USD)
PLA filament	For T-maze	26.39
PLA filament	For EPM	27.81
Epoxy resin	For structural reinforcement and sealing surface	12.00
Super glue	General assembly	2.00
Camera stand	For mounting EPM	8.00
Ender 3	3D printer	150.00
USB camera	For behavior tracking	68.00
Computer	Mini-PC for recording videos	400.00
	Total estimated cost	$694.20

We selected the Ender 3 printer for its affordability and strong open-source community support. However, many other low-cost printers are available. Slightly higher-end models, such as the Bambu Lab series ($800–$2,500), offer quality-of-life features like self-calibration, automatic bed leveling, and significantly faster print speeds. These features would significantly improve the experience of researchers setting up 3D printing for the first time. PLA is a popular choice for filament due to its ease of use and low cost. However, applications requiring increased durability or frequent cleaning may benefit for alternative materials such as PETG or ABS. These filaments offer greater strength, temperature resistance, and chemical tolerance, albeit with more demanding print settings.

The advantages of 3D printing extend beyond behavioral mazes. Object recognition tasks are widely used to assess cognitive function in rodent models (Leger et al., 2013). Using 3D-printed objects in the novel object recognition task offers significant benefits by providing standardized open-source designs (Inayat et al., 2021). Sharing print files addresses inconsistencies in object selection across laboratories, ensuring greater comparability of findings (Inayat et al., 2021). Traditionally, labs have used a variety of different objects (Leger et al., 2013). This diverse collection of items leads to variability in task difficulty, biases in exploration, and challenges in comparing results across studies (Gulinello et al., 2019; Inayat et al., 2021). The introduction of 3D-printed objects eliminates these discrepancies by allowing researchers to use identical objects, improving experimental rigor and reproducibility (Inayat et al., 2021). The same principles can be applied to the design of mazes and other behavioral apparatus. By sharing print files, researchers can ensure that environmental-apparatus variables remain consistent across studies. The ease with which design files can be shared make them a practical and scalable solution that enhances methodological consistency.

The use of neural networks in behavioral neuroscience has significantly advanced automated analysis, enabling precise tracking of behavior (Stern et al., 2015; Mathis and Mathis, 2020; Goodwin et al., 2024). Our study demonstrates the adaptability of convolutional neural network-based models, specifically the *EXPLORE* pipeline, which was originally developed for object recognition tests but was successfully retrained to analyze EPM behavior (Ibañez et al., 2023). This highlights the flexibility of deep learning models in behavioral research. By adjusting the training dataset, the same neural network architecture can be repurposed for different behavioral paradigms. One of the key strengths of this approach is its low computational demand (Ibañez et al., 2023). While deep learning is often associated with high-end GPU requirements, this implementation successfully operated on a CPU-based model, achieving reliable performance without the need for specialized hardware (Ibañez et al., 2023).

An emerging trend in the field is the development of generalized behavioral models, which could further reduce the need for individual researchers to train models from scratch (von Ziegler et al., 2021; Kolides et al., 2023; Ye et al., 2024). Since most behavioral studies rely on similar recordings, a foundational model trained on a wide variety of behaviors could provide pretrained neural networks that generalize across multiple experimental paradigms (Ye et al., 2024). These generalized models would eliminate the need for individual researchers to create and annotate their own datasets, significantly reducing the time and resources required to use deep learning-based analysis (Ye et al., 2024). As foundational models continue to evolve, the integration of pretrained networks for standardized behavioral assessments will further enhance accessibility and reproducibility in neuroscience research (Kolides et al., 2023). By demonstrating the utility of 3D-printed mazes and integrating them with machine learning-based tracking, our study aligns with a broader movement toward cost-effective, standardized, and scalable behavioral research. This approach expands research possibilities for established labs and lowers the barrier to entry for early-career scientists and institutions with limited funding.

## Synthesis

Reviewing Editor: Katrina Choe, McMaster University Decisions are customarily a result of the Reviewing Editor and the peer reviewers coming together and discussing their recommendations until a consensus is reached. When revisions are invited, a fact-based synthesis statement explaining their decision and outlining what is needed to prepare a revision will be listed below. The following reviewer(s) agreed to reveal their identity: Maithe Arruda-Carvalho, Raffaele Mazziotti.

This manuscript describes the integration of 3D-printing and machine learning for mouse behavioral testing, specifically in T-maze and elevated plus maze (EPM) paradigms. Reviewers commented on the utility of this method for researchers in terms of cost-savings and the potential for increased reproducibility. They also recommended several improvements to the manuscript, including more details on the tools and procedures, better documentation and a more realistic breakdown of costs. Their detailed reviews are copied below.

Reviewer 1

While their T-maze and EPM designs seem interesting, open-source designs with (more) detailed instructions for these types of mazes are already available (e.g. https://www.instructables.com/Building-a-Custom-Elevated-Plus-Maze-to-Conduct-Be/). Instead, the main strength of this manuscript would be to offer a detailed, one-stop-shop, integrated starter package for T-maze and EPM printing with behavioral analysis. As such, my main suggestion is that the authors reframe the narrative (and title) to express that, and offer a step-by-step open source guide for labs starting to use these apparatuses. This would include (1) adding far more detail on the printing and assembly of their mazes, including troubleshooting and more details on materials/hardware, and (2) offering a step-by-step guide to the implementation of the EXPLORE package for the use of the two mazes (which might need to be a collaboration with the original authors). Creating a thorough starter package that is as close as possible to plug-and-play for researchers just starting in this type of behavioral testing would be a valuable addition to the field. Below are more detailed suggestions:

- More detailed step-by-step printing and assembly instructions should be added for both mazes (e.g. how long should the epoxy be allowed to settle? How do you glue without having the maze stick to the ground? How do you remove excess? etc)

- As the authors do not see difference in anxiety-like behavior in the EPM, it would be important to discuss that limitation and/or offer data showing their strategy can capture changes in this behavior.

- Statistical comparison of the exploration time values obtained for each behavioral quantification methods would strengthen the manuscript, beyond correlation analyses.

- How do the authors deal with the segmentation of the maze? Commercial mazes are smooth - should researchers file any excess epoxy in each juncture to make the maze smoother? Do animals spend time sniffing or touching the junctures in the maze?

- Total cost savings argument should be adjusted as there are hidden costs associated with 3D printing, including the printer itself, in addition to the cost of the camera and computer necessary for the behavioral tracking (e.g. claims of 98% cost savings in line 234, as well as start of manuscript). While it is clear that this method is far cheaper than commercially available alternatives, the authors should be transparent and realistic about the actual cost of their proposal. Specifically, they should make those estimates with the maximum possible cost of their method, rather than the minimal the excludes all infrastructure. This avoids the risk of misleading early career researchers on the actual costs of this research. Also, the prices in table 1 do not match those cited in the discussion.

- Adding the actual hyperlink to the designs of the mazes (line 75) would ease access to readers

- Discussion of the best 3D printer filaments to use, as well as an extension of the discussion of alternative 3D printers would also help guide and make this more fail-proof for users

- Several technical details are missing in the methods, including (but not limited to) maze dimensions, camera specifics, EPM stand dimensions and availability.

- Fig 2C - Y axis label is missing

- Sentence in lines 200-201 is truncated

Reviewer 2

Title Revision

The current title is too generic and suggests a review rather than an experimental report. Please replace it with one that reflects the study's scope and findings, for example: "Low‑Cost 3D‑Printed T‑Maze and Elevated Plus Maze with Open‑Source ML Tracking for Mouse Behavior."

3D‑Parts Repository

The manuscript claims that STL files are available online but provides no link. A fully annotated GitHub (or better permanent repositories like Zenodo or NIH 3d) repository is essential for reproducibility; please include the exact URL or DOI.

Machine‑Learning Code Availability

No source code for the EXPLORE pipeline is supplied. The repository should contain at minimum:

- Video capture and preprocessing scripts

- Model training and inference code

- Example datasets and usage instructions

PLA Hygroscopicity

PLA absorbs moisture, which can degrade mechanical strength and surface finish over time. Please discuss how humidity and cleaning protocols might impact the durability and performance of printed mazes.

Comprehensive Cost Breakdown

The cost analysis covers only printed components. To allow fair comparison with commercial systems, include prices for:

- Video camera and lens

- Tripod or mounting hardware

- Lighting (visible or infrared)

- Computing hardware required for ML inference

Video‑Recording Rig Details

The Methods describe a generic "video camera" mounted on a tripod but omit critical specifics. Please specify:

- Camera model, resolution, frame rate, sensor type (IR vs. visible light)

- Cost of the camera and mounting system

- Any calibration procedures or settings

Expanded Introduction

The Introduction lacks depth on existing 3D‑printed tools in behavioral neuroscience. Please broaden this section to discuss prior applications (e.g., 3D‑printed object‐recognition tasks, operant chambers) and clearly position your work within that context.

Minor Points

Methods Structure

Enhance readability by subdividing the Methods into clear sections or numbered steps (e.g., "Maze Fabrication," "Assembly," "Video Acquisition," "Machine‑Learning Analysis").
